# Endoplasmic reticulum oxidoreductin 1α as a potential therapeutic target in diseases: from oxidative protein folding to pathophysiological mechanisms

**DOI:** 10.3389/fphar.2025.1709284

**Published:** 2025-11-17

**Authors:** Fang He, Xiaoyue Ge, Xiaohui Liu

**Affiliations:** 1 Affiliated Nanhua Hospital, Hengyang Medical School, University of South China, Hengyang, China; 2 The Academy of Chinese Health Risks of West China Hospital, Chengdu, China

**Keywords:** endoplasmic reticulum oxidoreductase 1α, cardiovascular diseases, cancer, diabetes, neurodegenerative diseases

## Abstract

Endoplasmic reticulum oxidoreductin 1α (ERO1α), an ER-resident thiol oxidoreductase, has been implicated in disulfide bond formation during protein folding by acting as an electron acceptor transfer for protein disulfide isomerase (PDI). This process reduces oxygen to H_2_O_2_ contributing up to 25% of the induced cellular reactive oxygen (ROS). However, research has shown that disulfide bond formation in certain proteins is preferentially catalyzed directly by ERO1, rather than indirectly through PDI. ERO1α also contributes to calcium homeostasis and endoplasmic reticulum stress (ERS). Disruption of these processes is closely associated with a variety of diseases, while the detailed molecular and cellular mechanisms underlying these processes remain to be elucidated. In mammals, tissue-specific ERO1α knockout and inhibitors have been developed to elucidate the cell-specific functions, but ERO1α inhibitors are not specific and may have significant cytotoxicity. This reviews provide an in depth summary regarding ERO1α in various disease processes, including cardiovascular diseases, diabetes, and cancer. Furthermore, it highlights the potential of ERO1α as a potential biomarker and a novel therapeutic target in clinical diseases.

## 1 Introduction

Since its initial discovery in mammals in 2000, ERO1α has sparked a multitude of speculations as to its functional significance. The most prevalent function of ERO1α is oxidative ER protein folding (Cabibbo et al., 2000). In eukaryotic cells, secretory proteins must undergo critical post-translational modifications within the endoplasmic reticulum (ER) including the formation of disulfide bonds-a process known as oxidative folding. This process highly relies on both the ER quality control system including folding enzymes and molecular chaperones, and its oxidizing environment of the ER maintained by a glutathione redox potential of (∼−200 mV) much higher than that in the cytoplasm (∼−300 mV) (Delaunay-Moisan et al., 2017). The chaperone PDI, required by over 30% of proteins for disulfide bonds formation, catalyzes disulfide bonds in nascent polypeptides by accepting electrons from free thiols (Shergalis et al., 2020). These electrons are then transferred to ERO1, which in turn reduces O_2_ to generate H_2_O_2_, the most well-understood ROS (Wang and chen, 2023).

ERO1 is flavin adenine dinucleotide (FAD) -dependent oxidase in the ER lumen which is involved in disulfide bonds formation (Shergalis et al., 2020). There are two members of the ERO1 family in mammals, the broadly expressed ERO1α (also known as ERO1 A or ERO1-L) (Cabibbo et al., 2000) and ERO1β (Pagani et al., 2000) which is specifically expressed in pancreatic and gastric cells (Dias-Gunasekara et al., 2005). Homozygous ERO1β mutant mice have a modest defect in oxidative folding of insulin and develop mild glucose intolerance (Zito et al., 2010). However, homozygous ERO1α mutant mice are superficially indistinguishable from wild-type mice and even compound homozygous ERO1α. Although in the hearts of the ERO1α mutant mice, ERO1β mRNA expression is increased, loss of function of both ERO1α and ERO1β is significantly heavier than in the ERO1α single knock model, which prompt ERO1β almost has no compensatory effect on myocardial calcium steady state (Chin et al., 2011), consistent with previous studies, there is nonredundancy of the ERO1α and ERO1β isoforms (Zito et al., 2010). Notably, unlike the embryonic lethality caused by ERO1 deletion in yeast, mice with loss-of-function mutations of ERO1α and ERO1β are viable and exhibit no discernible phenotypes which implicates the existence of redundant mechanisms for thiol oxidation (Zito et al., 2010; Chin et al., 2011).

Under pathological conditions such as chronic hypoxia (Chin et al., 2011) and metabolic disorders (Song et al., 2008) lead to accumulation of misfolded protein and ERS which elicit an adaptive response called the unfolded protein response (UPR). UPR is regulated by three ER sensors: inositolrequiring enzyme 1 (IRE1), protein kinase R (PKR)-like endoplasmic reticulum kinase (PERK), and activating transcription factor 6 (ATF6). If ERS persists, the ERS-related apoptotic pathway PERK/ATF4/C/EBP-homologous protein (CHOP) is activated (Chen et al., 2023). Then CHOP can significantly upregulate ERO1α expression (Song et al., 2008). The sustained overactivation of ERO1α results in excessive H_2_O_2_ production, which disrupts ER redox homeostasis and Ca^2+^ dysregulation. The Ca^2+^ dysregulation impairs the function of the sarcoplasmic reticulum (SR), the primary intracellular Ca^2+^ storage organelle, where Ca^2+^ is normally accumulated by sarco/endoplasmic reticulum Ca^2+^-ATPase2 (SERCA2) and released via major channels such as the ryanodine receptor (RyR) and the inositol 1,4,5-triphosphate receptor (IP3R). Ultimately, these processes accelerate disease development (Varone et al., 2021).

Thus, inhibiting ERO1α or its functional interaction with PDI has become an attractive therapeutic strategy. Although compounds like EN460 have shown efficacy in preclinical models, however, its off-target effect pose serious challenges for clinical application (Varone et al., 2025). This review aims to systematically review the pathophysiological roles of ERO1α in major human diseases including cardiovascular disorders, diabetes, cancer, and other diseases and discusses the associated challenges and future perspectives, with the goal of informing the development of novel ERO1α -targeted therapies.

## 2 Structure and function of human ERO1α

ERO1 was first discovered in 1998 by Frand and Kaiser in yeast (Frand and Kaiser, 1998). Subsequently, the human homolog ERO1-L was identified. ERO1-L is located on human chromosome 14 at q22.1. The predicted human gene product of ERO1-L is a 468-amino acid polypeptide which associates with N-glucoprotein and oxidative protein folding in the lumen of ER (Cabibbo et al., 2000). Inaba et al. have elucidated the crystal structures of both active and inactive forms of full length human ERO1α, revealing critical regulatory features in all eukaryotes. Human ERO1α modulates its oxidative activity by using Cys104 and Cys131 to regulate the mobility of the electron-shuttle loop; and its inactive form by the formation of Cys94–Cys131 and possibly Cys99–Cys104 disulphides yields (Inaba et al., 2010). But one recent study has showed no substantial conformation change between active and inactive forms of human ERO1α (Zhang et al., 2019).

**FIGURE 1:**
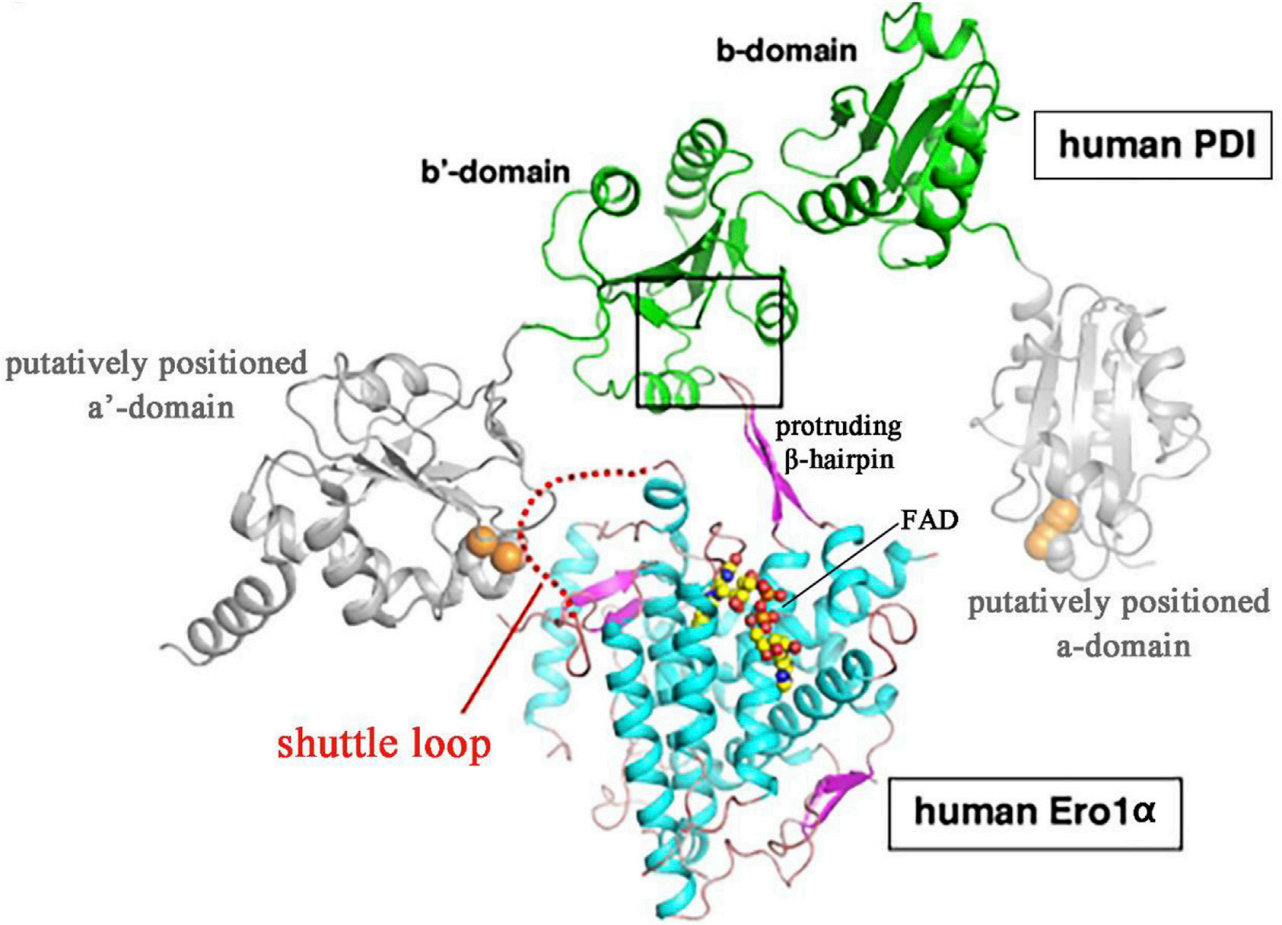
Predicted structures of ERO1α-PDI complex model (Wang et al., 2009; Araki and Inaba, 2012) Masui et al. used the website (sysimm.ifrec.osaka-u.ac.jp/surFit/index.html) to dock the full-length human ERO1α and the b-b’domain fragment of human PDI. The FAD molecule in ERO1 is depicted as small yellow-orange spheres. The square highlights the interface between the two enzymes. The purple hairpin sample part of human ERO1α is protruding β-hairpin. The shuttle loop is illustrated by a red dotted line.

Structural analysis has revealed that ERO1α selectively targets PDI through complementary electrostatic and hydrophobic interactions between its substrate-binding pocket and the b′ domain of PDI. PDI comprises four thioredoxin domains: two redox-active domains (the a and a′ domains), and two non-catalytic thioredoxin-like domains (the b and b′ domains) (Tian et al., 2006). Further studies revealed that a protruding β-hairpin structure in ERO1α engages with a hydrophobic pocket on reduced PDI’s b′ domain, thereby leading to preferential oxidation of the C-terminus PDI’s a′ domain (Masui et al., 2011) and resulting in the formation of a shuttle disulfide bond. The structure of human ERO1α features a four-helix bundle containing a FAD binding site, the flexible loop includes shuttle disulfide bonds which facilitate the transfer of electrons from PDI to the FAD prosthetic group (Wang et al., 2009) (Figure 1).

**FIGURE 2:**
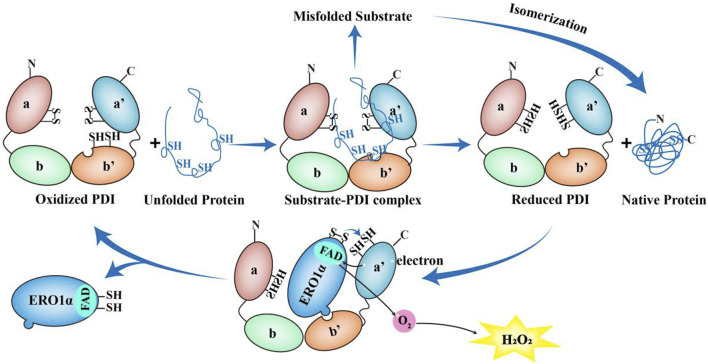
The role of ERO1α-PDI in the redox cycle. The unfolded proteins can bind to b' domain of the oxidized PDI to form the substrate-PDI complex. Then the unfolded proteins dissociate from PDI, resulting in the formation of native proteins and reduced PDI. Conversely, if misfolded substrate are formed, the reduced PDI or other members of the PDI family can function as isomerases to achieve the appropriate native proteins. After that, the reduced PDI is reoxidized by ERO1α. Once the protruding β-hairpin of ERO1α interacts with the hydrophobic pocket in b′ domain of PDI and the redox active site of PDI a'-domain is oxidized by ERO1α, the reduced PDI undergoes conformational changes and reduces its affinity for ERO1α and dissociates from ERO1α. Along with this process, ERO1α utilizes FAD subunit to transfer electrons from the a′ domain of the reduced PDI to molecular oxygen, thereby producing one molecule of H_2_O_2._ Then ERO1α dissociates from PDI and oxidized PDI starts a new round of protein folding oxidation.

ERO1α functions as an electronic exchange center for disulfide bond formation (Figure 2). In eukaryotic cells, the formation of disulfide bond depends on the oxidative environment of the ER which is driven by a high oxidized glutathione (GSSG)/reduced glutathione (GSH) ratio and requires correct protein folding for spatial proximity (Chen et al., 2024). ERO1α oxidizes PDI by transferring electrons via FAD, generating H_2_O_2_ as byproducts which generates up to 25% of induced cellular ROS, particularly in highly secretory cells (Zito, 2015). In addition, the UPR is triggered by ERO1α without PDI (zito, 2015). Additionally, ERO1α has been shown to promote Ca^2+^ release from the SR into the cytosol by regulating the IP3R and the RyR (Hamilton et al., 2022).

## 3 ERO1α and disease progression

Under pathological conditions including hypoxia and metabolic disorders, dysregulated ERO1α expression is closely related to the occurrence and development of various diseases such as cancer (Chen et al., 2024), neurodegenerative diseases (Lehtonen et al., 2016), cardiovascular disorders (Jha et al., 2023), diabetes (Wright et al., 2013), and inflammatory conditions (Ranchoux et al., 2015). The main mechanisms of disease occurrence and development can be summarized as follows: (1) Induction of cell apoptosis: ERO1α can cause ER oxidative stress by generating excessive ROS, which not only activates the apoptotic pathway of UPR such as ATF4/CHOP and disrupts calcium homeostasis, and induces inflammatory responses (Rao et al., 2015). Continuous activation of PERK leads to abnormal increase in ERO1α expression, generating more ROS, forming a vicious cycle, and ultimately promoting cell apoptosis and tissue damage (Bassot et al., 2023). ERO1α′s high expression is closely related to cell death in various diseases (Song et al., 2008). (2) Induction of Inflammation: ERO1α can promote the release of pro-inflammatory cytokines (Ranchoux et al., 2015). (3) Support tumor angiogenesis: In the tumor microenvironment, the expression of ERO1α may be regulated by factors such as hypoxia and the ROS and signals produced by it may affect endothelial cell function and promote angiogenesis (Xu et al., 2015). (4) Induce and enhance epithelial-mesenchymal transition (EMT), It produces ROS to create a permissive signaling environment that activates multiple key transcription factors and pathways which are central to initiating and sustaining the EMT (Yang et al., 2021). Therefore, high ERO1α expression is strongly associated with EMT and increased tumor aggressiveness, metastatic potential, and poor patient prognosis.

### 3.1 ERO1α and cardiovascular disease

Cardiovascular diseases mainly include arrhythmia, heart failure, stroke, etc. ERO1α drives oxidative stress and calcium homeostasis (Chin et al., 2011), and promotes plaque rupture (Zhang et al., 2021) and thrombosis (Jha et al., 2023), ultimately leading to serious consequences such as myocardial infarction and stroke.

**FIGURE 3:**
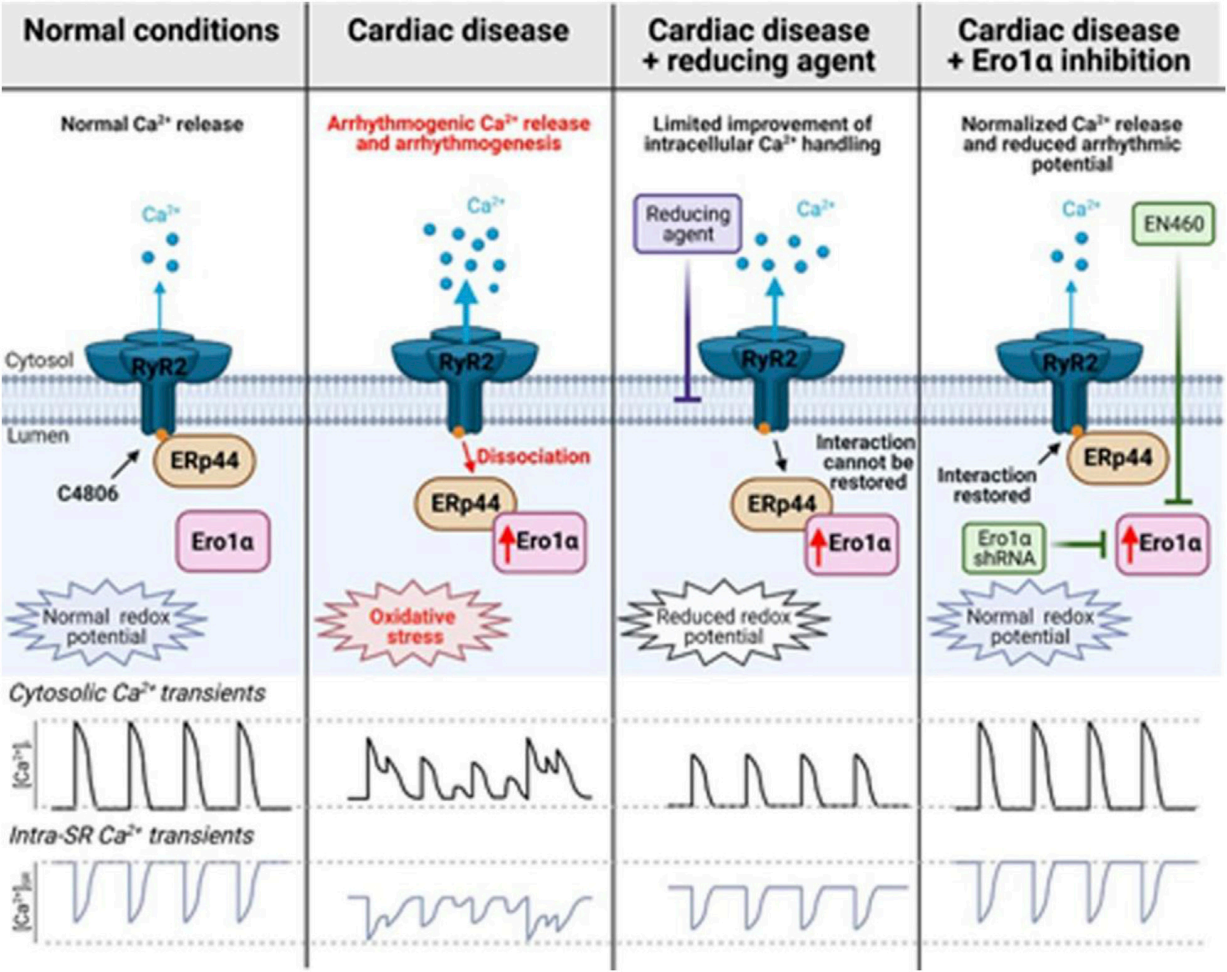
The role of ERO1α in ventricular arrhythmias in Ca^2+^-dependent hypertrophic hearts (Hamilton et al., 2022).

The myocardial RyR2 serves as the main channel for Ca^2+^ release from the SR, and hyperactivity of this process by posttranslational oxidative modifications may play a crucial role in Ca^2+^ dependent arrhythmogenesis in cardiac hypertrophy and failure (Morciano et al., 2022). ERO1α plays a crucial role in cardiac Ca^2+^ homeostasis. In homozygous ERO1α mutant adult cardiomyocytes, the peak amplitude of calcium transients was reduced and protected mice lacking ERO1α against progressive heart failure in a transaortic constriction model. However, the underlying molecular mechanisms remain unclear (Chin et al., 2011). In a thoracic aortic banding induced hypertrophic rat model, the upregulation of ERO1α heightened oxidative stress within the SR, leading to the disruption of the disulfide bond formed by the specific cysteine residue Cys4806 between the PDI protein endoplasmic reticulum protein 44 (ERp44) and RyR2, which resulted in the dissociation of ERp44 from the RyR2 complex, and increased the open probability of RyR2 and facilitated the spontaneous release of Ca^2+^. This mechanism contributes to a higher incidence of ventricular arrhythmias in Ca^2+^-dependent hypertrophic hearts. The small molecule inhibitor EN460 that can effectively inhibit ERO1α, reduced the oxidative state of the SR, enhanced the amplitude of Ca^2+^ transients and the Ca^2+^ content of the SR, and decreased spontaneous Ca^2+^ waves. Ultimately, this leads to a reduction in the incidence of ventricular arrhythmias in Ca^2+^-dependent hypertrophic hearts (Figure 3). In conclusion, ERO1α may represent a promising therapeutic target for mitigating arrhythmias and enhancing cardiac function in heart failure (Hamilton et al., 2022).

**FIGURE 4:**
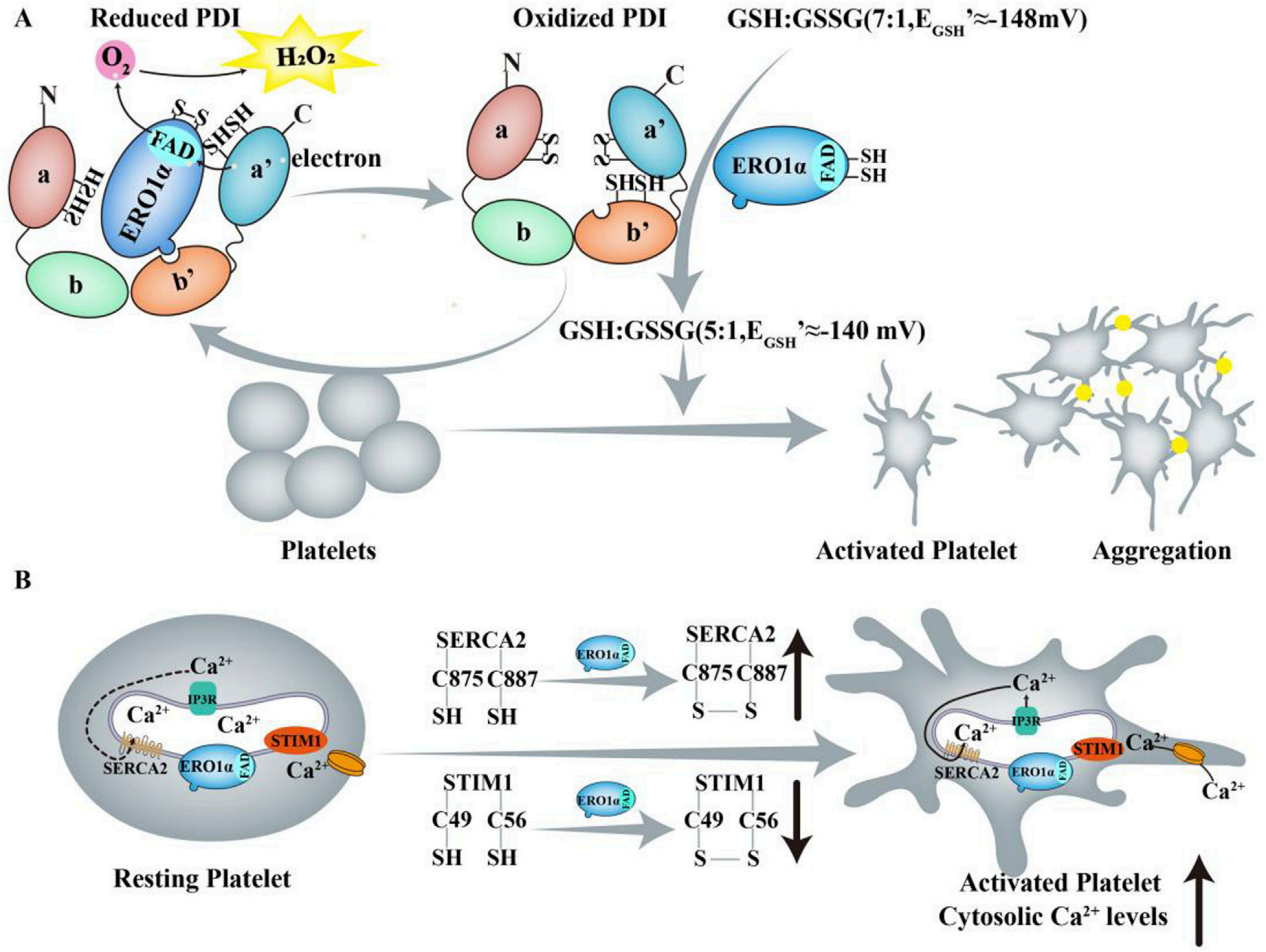
ERO1α and ERO1α/PDI regulate platelet function. **(A)** On the platelet surface, ERO1α continuously oxidizes PDI, thereby leaving the majority of PDI in an oxidized state. In this process, electrons are sequentially transferred from GSSG to PDI, ERO1α and finally to O_2_. ERO1α and PDI are released from platelets surface into the plasma which further oxidizes GSH to GSSG, thereby altering the GSH/GSSG ratio and increasing the reduction potential of GSH from −148 mV (GSH/GSSG≈7:1) to approximately −140 mV (GSH/GSSG≈5:1) which provides an optimal reduction potential for platelet aggregation and allows platelets to go from an inactive to an activated state. **(B)** Upon agonist stimulation, ERO1α may interact with IP3Rs to induce Ca^2+^ release. ERO1α modified an allosteric Cys49 - Cys56 disulfide bond in STIM1 and a Cys875 - Cys887 disulfide bond in SERCA2, resulting in an increase in the binding of ERO1α to STIM1 and, conversely, a decrease in the binding of ERO1α to SERCA2 which contribute to the Ca^2+^ store content.

Thrombotic and thromboinflammatory diseases, including atherothrombosis and ischemic stroke, are characterized by increased platelet activity (Schanze et al., 2019; Stoll and Nieswandt, 2019). Following arterial injury, platelets adhere to collagen and von Willebrand factor and aggregate through the interaction of fibrinogen and activated αIIbβ3 integrin, resulting in vaso-occlusive thrombosis. The mechanisms of hemostasis following tissue damage are analogous. Consequently, platelets play a crucial role in both thrombosis and hemostasis (Li et al., 2010). Confocal microscopy and biochemical studies have demonstrated that ERO1α colocalizes with PDI and αIIbβ3 on the surface of unstimulated platelets (Swiatkowska et al., 2010). On the platelet surface, ERO1α continuously oxidizes PDI, resulting in altering the GSH/GSSG ratio and increasing the reduction potential of GSH from −148 mV to approximately −140 mV which enzymes an optimal reduction potential for platelet aggregation (Figure 4A) (Wang et al., 2022). However, other studies have demonstrated that ERO1α is neither released nor detected on the platelet surface but is localized within the dense tubular system, a Ca^2+^ storage organelle. Furthermore, ERO1α in platelets does not regulate PDI activity as previously hypothesized. The intracellular ERO1α in a PDI- independent manner influences Ca^2+^ store content and Ca^2+^ mobilization by altering the Cys49-Cys56 disulfide bond in stromal interaction molecule 1 (STIM1) and the Cys875-Cys887 disulfide bond in SERCA2, resulting in platelet activation and aggregation (Figure 4B) (Jha et al., 2023). The deletion of ERO1α or inhibition with a small-molecule inhibitor B12-5 (IC_50_ = 7.9 μM) results in reduced infarct volume and improved neurological outcomes in ischemic stroke models, indicating that targeting ERO1α may offer potential benefits in stroke treatment. But B12-5 is likely to inhibit the activity of both ERO1 isoforms (ERO1α and ERO1β) and diminishes monoamine oxidase A (MAO-A) activity. M6766 (IC_50_ = 1.4 µM) shows >70-fold selectivity over compared to other tested enzymesis such as PDI and MAO-A. Although it also inhibits ERO1β, studies using megakaryocyte-specific ERO1β conditional knockout mice suggest that ERO1β is not affect platelet activation and aggregation. However, the deletion of ERO1α/β does not affect the basal level of platelet activation and targeting ERO1 in non-platelet cells may lead to off-target affects (Wang et al., 2025). Overall, selectively targeting ERO1α represents a promising approach for preventing or treating thrombotic diseases.

#### 3.1.1 Controversy: ERO1α localization in platelets

Conflicting findings exist regarding whether ERO1α is surface-exposed or localized intracellularly in platelets. On the one hand, Swiatkowska et al. (2010) reported surface localization using subcellular fractionation, confocal microscopy and flow cytometry in resting platelets, but their approaches relied on exogenous recombinant ERO1α and antibody labeling, which may detect adsorbed protein rather than endogenous ERO1α. Moreover, no secretion mechanism was demonstrated. In contrast, Jha et al. (2023) used intravital microscopy and immunogold electron microscopy which has a higher resolution to show that ERO1α is not present on the platelet surface, but rather localized exclusively within the dense tubular system in resting and activated human platelets. On the other hand, Swiatkowska et al. used resting platelets which are mostly used *in vitro* studies, while Jha et al. employed intravital microscopy combined with fluorescently labeled non-blocking polyclonal anti-ERO1α antibodies to detect extracellular ERO1α at the site of laser-induced cremaster arteriolar injury in mice. This result suggests that platelets are unlikely to be the main source of extracellular ERO1α. Therefore, current evidence favors intracellular, dense tubular system-restricted localization of ERO1α in platelets.

**FIGURE 5:**
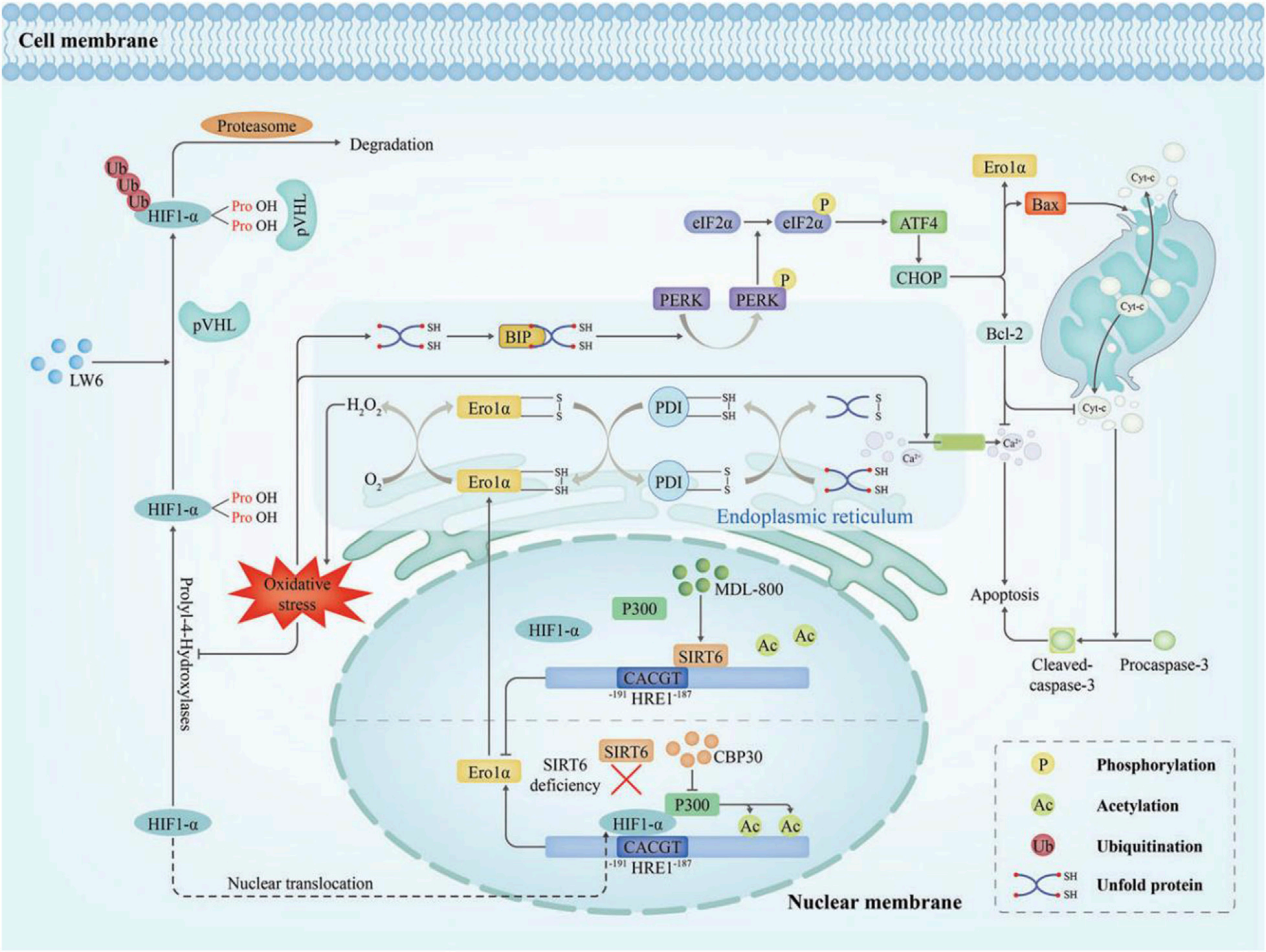
Diagram of the molecular mechanism of Ero1α in I/R injury (Bassot et al., 2023). I/R injury evokes oxidative stress that drives HIF1α into the nucleus, where it upregulates ERO1α transcription. Over time, ERO1α-PDI generated excessive H_2_O_2_ which disrupted ER redox homeostasis, increased Ca^2+^ efflux from the ER triggering apoptosis, and burdened the ER with unfolded protein. As a result, the overload of unfolded proteins, the overactivation of ERS and maladaptive UPR can occur. The PERK-eIF2α-CHOP signalling pathway was activated and precipitated apoptosis. MDL-800 activates the deacetylase activity of SIRT6, then SIRT6 deacetylates H3K9 at the ERO1α promoter which is responsible for inhibiting HIF1α/p300-mediated ERO1α transcription.

Early reperfusion is crucial for recovery following acute myocardial infarction (MI). However, it can also induce ischemia/reperfusion (I/R) injury (Braunwald and Kloner, 1985). But the pathophysiological mechanisms underlying I/R injury remain incompletely understood. Research indicated that ERO1α expression was upregulated in H9C2 cardiomyocytes under the hypoxia/reperfusion (H/R) which effectively simulate myocardial I/R injury, and reaching their peak after 3 h of hypoxia and 6h of reoxygenation. Furthermore, ERO1α knockdown with ERO1α- shRNA had been shown to decrease ERS and apoptosis in myocardial cells after H/R injury by inhibiting intracellular ROS production and decreasing intracellular Ca^2+^ levels (Lai et al., 2020). However, the results *in vitro* cannot fully reflect those *in vivo* and further research is required. The expression of ERO1α during I/R can be regulated by Sirtuin6 (SIRT6) (Guo et al., 2023). SIRT6 is an NAD^+^-dependent protein that protects endothelial cells from inflammatory and oxidative stress damage (Guo et al., 2022). It was observed that SIRT6 deacetylates H3K9 to block the recruitment of hypoxia-inducible factor 1α (HIF1α) and p300 at the ERO1α promoter, thereby suppressing ERO1α expression in cardiac microvascular endothelial cells exposed to oxygen–glucose deprivation/reperfusion. In addition, following ischemia‒reperfusion surgery, ecSirt6^−/−^ mice exhibited upregulation of ERO1α expression (Figure 5) (Guo et al., 2023).

Hyperhomocysteinemia (HHcy) is a risk factor for cardiovascular diseases such as stroke, myocardial infarction, and arterial and venous thrombotic events (Pernaa et al., 2003). X. Wu et al. found via Western blot that ERO1α was upregulated approximately sixfold in blood vessels of HHcy mice, and immunofluorescence staining showed that Ero1α was located in thoracic aortas of HHcy mice (Wu et al., 2019). Consistent with these results, ERO1α was also upregulated in macrophages within plaques of HHcy atherosclerosis mice fed a high Hcy diet (1.8 g/L) and mouse peritoneal macrophages compared with control (Zhang et al., 2021). The mechanism involves Hcy facilitating the binding of HIF1α to the ERO1α promoter, thereby enhancing its transcriptional activity. Moreover, Hcy upregulates the GSH/GSSG ratio resulting in a markedly increase in H_2_O_2_ levels and the ER oxidative stress in endothelial cells (Wu et al., 2019). In the context of atherosclerosis, the development of vulnerable plaques in atherosclerosis promoted by Hcy upregulates ERO1α expression and activates ERS-dependent macrophage apoptosis (Zhang et al., 2021). In conclusion, ERO1α was upregulated in Hcy-induced cardiovascular pathology and may be related to ERS.

### 3.2 ERO1α and diabetes mellitus

Diabetes is a chronic metabolic disorder characterized by pancreatic β-cell dysfunction and insulin resistance. Pancreatic β-cell dysfunction leads to insufficient insulin secretion and disrupted glucagon regulation, while insulin resistance reflecting diminished responsiveness of peripheral tissues to insulin. It is primarily classified into type 1 and type 2 diabetes (T2DM). Type 1 diabetes results from an absolute deficiency of insulin, while T2DM stems from insulin resistance accompanied by a progressive decline in β-cell function (American Diabetes Association Professional Practice Committee, 2025).

Proinsulin is synthesized and secreted by pancreatic β cells and is a precursor to insulin. Proinsulin misfolding is an important cause of pancreatic β -cell failure and diabetes. ERO1α and ERO1β play a key role in the oxidative folding and secretion of proinsulin (Sun et al., 2015). Zito et al. (2010) suggest that the deficiency of ERO1-β can impair oxidative folding and proinsulin maturation and eventually cause diabetes using homozygous ERO1β mutant mice and ERO1β deficient Min6 cells. Surprisingly, their are nonredundancy of the ERO1α and ERO1β isoforms. This suggests that in mammals, there may exist an ERO1-independent disulfide bond formation mechanism, especially in non-pancreatic tissues. However, Wright et al. (2013) in Mutant Ins-gene-induced Diabetes of Youth show that the overexpression of ERO1α has been shown to rescue secretion of mutant proinsulin-G (B23) V misfolding which is defective in formation of native disulfide bonds and limiteds the ERS, but this study did not address isoform redundancy, and lacked validation *in vivo*. As shown by Zito et al. (2010), *in vitro* and *in vivo* outcomes can diverge: in cultured Min6 cells, compromise of ERO1-β improved their ability to cope with enforced expression of the mutant misfolded proinsulin Akita, but this benefit was not realized in mutant mice. These differences may stem from higher O_2_ in culture, absent autocrine insulin signaling, divergent death pathways, and developmental effects that erase the “reducing-ER” benefit seen in cells. Therefore, the role of ERO1α in proinsulin folding remains to be firmly established.

In most cells, H_2_O_2_ produced by ERO1 can be cleared by glutathione peroxidase 8 and peroxidase reductase 4 within the ER lumen (Yoboue et al., 2018). However, pancreatic β cells hardly express glutathione peroxidase 8 and have extremely low levels of peroxiredoxin 4 thus being particularly sensitive to H_2_O_2_ derived from ERO1 (Mehmeti et al., 2017). Studies have shown that CHOP knockout can reduce the expression of ERO1α, alleviate oxidative stress, improve islet β cell function and promote cell survival in a variety of diabetic mouse models, thereby slowing the progression of T2DM. Future studies should focus on whether Chop deletion protects β cells from oxidative damage through the downregulated expression of ERO1 (Song et al., 2008). Studies have shown that palmitic acid, a non-esterified saturated fatty acid derived from adipose tissue, plays a key role in the development of T2DM. Palmitic acid can lead to apoptosis of pancreatic β cells by inducing oxidative stress and ERS. Although both ERO1 subtypes are expressed in β cells, palmitate specifically induces the expression of ERO1α only. Specific knockdown of ERO1α can significantly alleviate ER oxidative stress caused by palmitic acid, reduce the accumulation of H_2_O_2_ in the lumen and mitochondria, restore ER calcium homeostasis, and improve mitochondrial membrane potential and ATP levels, thereby effectively inhibiting palmitic acid-induced β-cell death. Therefore, increasing the expression of ERO1α may promote β -cell apoptosis (Sharifi et al., 2024).

Adiponectin is secreted by adipocytes, exhibits protective effects against insulin resistance, T2DM, and cardiovascular disease (Achari and Jain, 2017). Research has demonstrated that the secretion of the adiponectin oligomers is tightly controlled by a pair of molecular chaperones including ERp44 and ERO1α in the ER. Upregulation of ERO1α enhances the secretion of adiponectin trapped by ERp44 (Wang et al., 2008). The covalent binding between ERp44 and ERO1α may be controlled by modulating ERp44 SUMOylation in adipocytes which provides a viable strategy for addressing obesity and insulin resistance. In adipocyte-specific Ubc9 deficient mice fed a high-fat diet exhibiting obesity, insulin resistance, and hepatosteatosis, the deficiency of Ubc9 results in the loss of ERp44 SUMOylation at lysine 76 (K76) in the thioredoxin-like domain. This alteration enhances ERp44 degradation and inhibits its covalent binding to ERO1α, thereby promoting ERO1α secretion and reducing ERS and inflammatory responses (Xie et al., 2023). Therefore, increasing the expression of ERO1α is a possible strategy for the treatment of diabetes and diabetic insulin resistance. But the upregulation of ERO1α expression may promote β-cell apoptosis posing a potential risk to islet function.

### 3.3 ERO1α and cancer

Cancer is characterized by uncontrolled cell proliferation, invasion and metastasis.

The expression of ERO1α is significantly upregulated in a variety of cancers and associated with poor prognosis. By regulating oxidative folding, ERS, Vascular endothelial growth factor (VEGFin hypoxia, ERO1α promotes tumor growth, angiogenesis and metastasis and chemoresistance) (Varone et al., 2021). It also enables immune escape via PD-L1 surface regulation (Chen et al., 2024). Consequently, ERO1α inhibition represents a promising strategy for selective tumor cytotoxicity with minimal toxicity to normal cells.

Studies have found that ERO1α is more correlated with cancer than ERO1β. Andrea G. Shergalis et al. analyzing the Cancer Genome Atlas data (Lee et al., 2015), found that ERO1α was continuously and actively expressed in a cohort of cancer patients in 33 diseases. Median patient ERO1α expression was higher than median patient ERO1β expression in 28 of 33 diseases (85%) (Wang and Chen, 2023). The high expression of ERO1α is associated with reduced survival rate and disease progression (tumor stage, grade, or glioma type) in cancer patients, indicating that the expression of ERO1α can be a prognostic factor for patients with cancer, including cervical cancer (Zhang et al., 2019; Huang and LI, 2021), cholangiocarcinoma (Yan et al., 2019), gastric cancer (Seol et al., 2016), breast cancer (Varone et al., 2021; Tanaka et al., 2016; Kutomi et al., 2013), lung cancer (Kim et al., 2016; Endoh et al., 2004)_,_ hepatocellular carcinoma (Yang et al., 2018), colorectal cancer (Wu et al., 2023) and multiple myeloma (Hayes et al., 2019). ERO1α can also be used as a new endogenous marker of chronic hypoxia, more reliable than carbonic anhydrase 9, a diagnostic biomarker and therapeutic target for cancer (Takei et al., 2019).

ERO1α directly promotes tumor phenotypes by regulating multiple signaling pathways. Tumor hypoxia and ERS are important drivers of tumor progression and metastasis. Under these conditions, ERO1α is upregulated and promotes breast cancer cell metastasis through oxidative folding-mediated activation of VEGF-A and ATF4/CHOP/VEGF pathway (Varone et al., 2021). EMT is an important biological process for tumor cells to metastasis and invasion. Studies have shown that ERO1α serves as a central regulator of EMT enhancing invasion and metastasis in cancers such as hepatocellular carcinoma (Yang et al., 2021). Its EMT-promoting function is further demonstrated in cervical cancer via ERO1α-PDI-induced ROS generation (Zhang et al., 2019). in lung cancer through IL-6/sIL-6R signaling and mucin-16 modulation (Ranchoux et al., 2015), and in cholangiocarcinoma via the Akt/mTOR pathway (Yan et al., 2019). Beyond EMT, ERO1α enhances cell proliferation and suppresses apoptosis in colorectal cancer by activating the PI3K/AKT pathway (Wu et al., 2023), supports gastric tumor progression through Akt and JNK signaling (Seol et al., 2016), and in pancreatic cancer, activates the Wnt/β-catenin axis to stimulate migration, invasion, and proliferation (Takei et al., 2019; Takei et al., 2017). Additionally, ERO1α induces ferroptosis resistance and contributes to carcinogenesis in mTORC1-activated cells by stimulating the transcription of solute carrier family 7 member 11 and activating the IL-6/signal transducer and activator of transcription 3 (STAT3) pathway (Wang et al., 2024).

Angiogenesis plays a crucial role in tumor growth, invasion and metastasis. VEGF is a key regulator of angiogenesis (Xu et al., 2015). ERO1α expression is upregulated by HIF1α to enhance protein secretion under hypoxia conditions and may inhibit tumor growth by targeting VEGF-driven angiogenesis (May et al., 2005). In the human breast cancer cell line MDA-MB-231, ERO1α promoted angiogenesis by facilitating the oxidative folding of the VEGF protein and enhancing VEGF mRNA expression. This is corroborated by clinical data in triple negative breast cancer cases, where the expression of ERO1α is correlated with the number of blood vessels (Tanaka et al., 2016). In addition to the finding that ERO1α deficiency can inhibit angiogenesis by increasing N-glycosylation of VEGFA (Varone et al., 2022), ERO1α also induces the S1PR1/STAT3/VEGF-A signaling pathway triggers tumor migration, angiogenesis, invasion, and EMT of hepatocellular carcinoma (Yang et al., 2018).

Tumor-associated immunosuppression, especially via programmed cell death ligand 1 (PD-L1) and other checkpoint molecules, enables tumors to evade immunity and co-opt the immune system for their own growth. PD-L1 binding to programmed cell death-1 on immune cells induces T cell apoptosis and tolerance. In breast cancer, ERO1α enhances the expression of PD-L1 by facilitating oxidative protein folding and augmentation of HIF1α; conversely, depleting ERO1-α inhibited apoptosis of PD-1+ T cells (Tanaka et al., 2017). Beyond PD-L1, ERO1α contributes to immune evasion under hypoxia by altering the redox state and surface expression of MHC class I in colon cancer, thereby alteration of susceptibility by CD8^+^ T cells (Kukita et al., 2015). Additionally, in breast cancer, ERO1α inhibits the T cell response by recruiting myeloid-derived suppressor cells (Tanaka et al., 2015). However, this study did not clarify the mechanism by which ERO1α regulates host immunity. Recent studies have demonstrated that ERO1α deletion results in an imbalance between IRE1α and PERK signaling pathways and triggers the lethal UPR. This process stimulates immunogenic cell death, thereby promoting host antitumor immunity. Notably, ERO1α-deficient tumors are more sensitive to PD-1 blockade (Liu et al., 2023). Therefore, ERO1α has a complex role in immunosuppression and potential as a target for tumor immunotherapy.

EN460 is a nonselectivity ERO1α inhibitor, but its potential off-target effects exclude it from clinical use. E. Varone et al. have been identifed two new EN460 analogs (I2 and I3), which efficiently inhibited ERO1α and blunted VEGFA secretion. Among them, I2 (IC50 = 8. 1μM) more potently suppressed cancer-relevant phenotypes, including VEGFA secretion and cell migration. Furthermore, I2 demonstrated efficacy in modulating the tumor microenvironment and impairing the viability of triple-negative breast cancer xenografts and syngeneic models. These findings establish the feasibility of pharmacological ERO1α inhibition and highlight its promise as an effective therapeutic strategy for the currently incurable triple-negative breast cancer (Varone et al., 2025).

### 3.4 ERO1α and other diseases

Beyond its established role in cancer, ERO1α is critically implicated in the pathogenesis of neurodegenerative diseases, aging, and hereditary myopathies, primarily through mechanisms of oxidative proteostasis. In neurodegenerative diseases such as Parkinson’s disease and Alzheimer’s disease, the ER protein misfolding under excessive stress can activate ERO1α, exacerbating cell oxidative stress and further damage to neurons (Zito, 2015). In the 1-methyl-4-phenylpyridinium (MPP^+^) model of Parkinson’s disease, inhibition of PDI or inhibition of ERO1α by EN460 (IC_50_ of 1.9 μM) can prevent accumulation of α-synuclein and MPP^+^ neurotoxicity in the ER. However, it is not clear whether ERO1α directly or indirectly modulates a-synuclein accumulation via its actions on PDI (Lehtonen et al., 2016). No published studies have focused on the contributions of ERO1α to the pathogenesis of Alzheimer’s disease. A case report involving a Finnish patient with progressive neurodegenerative disease leukoencephalopathy - caused by a novel homozygous mutation in *Hikeshi* - revealed a significant reduction in ERO1α expression in the patient’s fibroblasts. This decrease may result from impaired heat shock response and ERS caused by defects in Hikeshi protein (Vasilescu et al., 2017). Cognitive dysfunction is the core symptom of neurodegenerative diseases. In hypoxia-induced cognitive dysfunction, the upregulation of ERO1α may lead to an increased production of ROS, which in turn affects mitochondrial function and apoptosis and impairs cognitive function (Zhang et al., 2022). Therefore, ERO1α is closely related to neurodegenerative diseases, but the specific regulatory mechanism of it remains unclear.

ERO1α is also involved in the regulation of aging. ERdj5, a disulfide reductase in the ER, maintains the redox homeostasis of the ER and combats oxidative stress during aging by receiving electrons from ERO1α and inhibiting the ERO1α-dependent production of H_2_O_2_ in the ER (Uegaki et al., 2023). Nitric oxide generated by activated inducible Nitric oxide synthase reacts with specific cysteine residues (e.g., Cys166) of ERO1α which leads to the S-nitrosylation of ERO1α and reduces the production of oxidants in the ER, thus causing ER reducing stress and finally promoting the aging process of cells (Qiao et al., 2022).

ERO1α may be a potential therapeutic target in hereditary myopathies (Roy et al., 2024). Selenoprotein N1 (SEPN1) is a protein located in the ER, and its genetic defects can lead to selenoprotein N-associated myopathy (SEPN1-RM), an inherited disorder that causes muscle weakness and respiratory failure. We found that ERO1α expression was upregulated in SEPN1 knockout cell model, and loss of SEPN1 impaired ER redox homeostasis and short-distance mitochondria-associated ER membranes, affecting Ca^2+^ dynamics between ER and mitochondria, and subsequently affecting muscle cell bioenergetics and muscle contractile function. Inhibition of ERS by the chemical molecular chaperone tauroursodeoxycholic acid (TUDCA, a ubiquitously ERS inhibitor) or inhibition of ERO1α by the inhibitor EN460 ameliorated the myopathic phenotype caused by SEPN1 deficiency (Germani et al., 2024). SELENON is also an ER protein, and its gene dysfunction can also cause muscle dysfunction. Deficiency of SELENON alters activity-dependent calcium handling, affects Ca^2+^ absorption of SR, and triggers ERS. These include maladaptive CHOP-induced ERO1. CHOP knockdown in SELENON knockout mice can reduce ERO1α expression and restores Ca^2+^ absorption, completely prevents diaphragm dysfunction, and prolongs the relaxation time of limb muscles after fatigue (Pozzer et al., 2019).

ERO1α is also associated with human viral infectious diseases, such as hepatitis C virus (HCV) and coronavirus. We showed that ERO1α expression was upregulated by HCV core protein, and knockdown of HCV core protein not only reduced cytoplasmic H_2_O_2_ levels but also blocked Ca^2+^ -monopherin-induced ROS production in mitochondria. In contrast, ERO1α expression was unchanged in NS5A-overexpressing cells. Therefore, HCV core protein may affect the oxidation state of cells by inducing ERO1α, while NS5A protein does not directly affect the expression of ERO1α, suggesting that different protein components of HCV may affect the oxidation balance of host cells through different mechanisms (Smirnova et al., 2016). The spike (S) protein of SARS-CoV-2 requires the formation of disulfide bonds for proper folding and trimerization, and ERO1α may reduce its binding to the host receptor by destroying the key disulfide bond of S protein, thereby weakening the infectivity of the virus (Yamaoka et al., 2022).

In addition, ERO1α is also associated with Hcy-induced nonalcoholic fatty liver disease, liver injury, and acute liver failure. nonalcoholic fatty liver disease is a chronic liver disease characterized by the accumulation of triglycerides in the liver. nonalcoholic fatty liver disease is associated with increased Hcy levels. In HHcy C57BL/6 mouse model cultured with high methionine diet or drinking water supplemented with Hcy (1.8 g/L) and blood samples collected from HHcy, researchers found that Hcy promoted H_2_O_2_ accumulation by activating the HIF1α-ERO1α pathway, causing ER peroxidation and ERS. In turn, the lipolysis reaction is activated to promote steatosis of liver cells (Yan et al., 2020). However, in homocysteine-induced liver injury, Hcy promotes ERS and apoptosis of hepatocytes by inhibiting the expression of ERO1α (Shen et al., 2022). Also in acute liver failure, CHOP increases ROS levels and promotes ERS by activating ERO1α, thereby exacerbating liver injury during acute liver failure (Rao et al., 2015).

## 4 Inhibitors of ERO1α

Consequently, inhibiting the ERO1α-PDI interaction and developing selective ERO1α inhibitors present promising avenues for treating related diseases, while a limited number of inhibitors are available for further validation and none are approved for clinical application. The main obstacle is the high structural conservation of the FAD-binding domain, ERO1α inhibitors also recognize other FAD-dependent enzymes like lysine-specific demethylase 1 (LSD-1) and MAO-A/B (Ranchoux et al., 2015).

Bisphenol A, an endocrine disruptor, has been shown to significantly inhibit ERO1-PDI-mediated disulfide bond formation by obstructing the substrate-binding pocket in the b' domain of PDI and the phenol groups of Bisphenol A compete for a highly conserved tryptophan residue within the protruding β-hairpin of ERO1α (Okumura et al., 2014).

A high-throughput *in vitro* assay revealed that EN460 (IC_50_ = 1.9 µM) selectivity inhibited ERO1α activity *in vivo* as well as *in vitro* by forming an adduct with the thiol group of ERO1α, resulting in the release of the FAD prosthetic group. Despite EN460 exhibiting nonselective reactivity towards free thiols, it specificity inhibited ERO1α. Another inhibitor QM295 (IC_50_ = 1.9 µM) whose structures also suggest the ability to react with free thiols had a less significant oxidation effect and toxicity on ERO1α than those of EN460 (Blais et al., 2010). Thus, EN460 extensive uses as a pharmacological validation tool for ERO1α. However, K.E. Hayes, et al. has shown it inhibits other flavoenzyme family members including LSD-1, MAO-A/B (Ranchoux et al., 2015). Then Brennan et al. identified an ERO1α inhibitor T151742 (IC_50_ = 8.27 µM) showed no detectable binding to the FAD-containing enzyme LSD-1 and was more sensitive than EN460 (IC_50_ = 16.46 µM), but it still inhibits MAO-A/B (Johnson et al., 2022). Recently, Jha et al. reported an ERO1α inhibitor B12-5 (IC_50_ = 8 µM) which reduced platelet activation and aggregation *in vitro* and platelet thrombus formation *in vivo*, but also reduced the activity of MAO-A (Jha et al., 2023). Then WANG J et al. identified a selective inhibitor of ERO1α named M6766 (IC_50_ = 1.4 µM) through a high-throughput screen of 39,901 compounds. M6766 binds to the FAD-binding pocket in ERO1α, exhibits 5-fold greater potency for except ERO1β (IC_50_ = 7.2 µM) and >70-fold selectivity over Mao-A and PDI. Nevertheless, M6766 has limited solubility and targeting ERO1 in non-platelet cells may lead to unexpected side effects (Wang et al., 2025). I2 (IC_50_ = 3.5 µM) and I3 (IC_50_ = 8.1 µM) specifically inhibits the redox activity of ERO1α by covalently binding to the Cys397 site of ERO1α. while it did not cause widespread oxidative stress or off-target effects. The toxicity of I2 is lower than EN460 and I3 and its tolerance *in vivo* is better. It performs more effectively in inhibiting VEGF secretion and migration. Therefore, I2 is more suitable as a candidate drug for further development (Varone et al., 2025) (Table 1).

**TABLE 1:** Summary Inhibitors of ERO1α.

Inhibitor	IC_50_ (μM)	Selectivity and toxicity	Reference
EN460	1.9	Nonselective reactivity with free thiols, and inhibit multiple flavoenzyme family members including LSD-1, MAO-A, and MAO-B. Toxicity at the highest concentrations	Ranchoux et al. (2015), Blais et al. (2010)
QM295 T151742	1.9 8.27	The specificity is still unclear. Selectivity for ERO1α over ERO1β or other flavoenzymes, but inhibits MAO-A/B	Blais et al. (2010), Johnson et al. (2022)
B12-5	8	Nonselective reactivity with other FAD-binding enzymes, but inhibits ERO1β and MAO-A	Jha et al. (2023)
M6766	1.4	Exhibits >70-fold selectivity over other tested enzymes, but inhibits ERO1β with an IC_50_ of 7.2 μM	Wang et al. (2025)
I2/3	3.5/8.1	Specific inhibition of ERO1α. Toxicity of I2 is lower than EN460 and I3, with good tolerance *in vivo*.	Varone et al. (2025)

However, there are still multiple challenges for the development of selective ERO1α inhibitors, in addition to the conservation of the FAD domain. 1. The structure of ERO1α shares 65.4% amino acid identity with ERO1β (Ranchoux et al., 2015). 2. Small molecule inhibitors need to effectively enter the ER cavity to exert their effects. 3. Off-target effects: Ensure specificity for other intracellular redox enzymes. 4. Verify its efficacy and safety in complex physiological environments.

## 5 Conclusion

ERO1α plays an important role in the maintenance of ER redox homeostasis and ERS that has been associated with a variety of diseases, but the exact mechanism of ERO1α in different diseases is still controversial. It has not been determined whether high expression of ERO1α in cancer directly promotes tumor progression or is merely a marker of changes in the tumor microenvironment. Future studies may reveal more about the regulatory mechanism of ERO1α and how to treat related diseases by targeting ERO1α.

Research on the role of ERO1α inhibitors in clinical diseases may provide new ideas and directions for the prevention of clinical diseases and the development of drugs. ERO1α inhibitors such as EN460, QM295, T151742, more or less have reactivity with free thiols and multiple flavoenzyme family members including LSD-1, MAO-A, and MAO-B. Even if M6766 does not exhibit antioxidant effects or thiol reactivity up to 100 μM and inhibit MAO-A, but it inhibits ERO1β with an IC50 of 7.2 μM. Inhibiting ERO1β may lead to unexpected adverse effects and obscure the therapeutic benefits of selectively blocking ERO1α. Therefore, it is necessary to develop highly specific ERO1α inhibitors. I2 is a specific inhibitor of ERO1α. It exhibits anticancer activity and low toxicity, however, its clinical potential remains uncharacterized.

The potential of ERO1α as a predictive and prognostic biomarker in cancer requires further validation and methodological standardization. The major difficulty is in the clinical detection and quantification of ERO1α. The current research mainly relies on technologies such as RNA-seq, immunohistochemistry technology and single-cell sequencing to identify biomarkers, which is complex and not suitable for clinical testing. The central challenge is reconciling serum-based detection with tissue-specific expression. While measuring ERO1α in patient serum would be ideal for diagnostics and monitoring, it is unclear if it is reliably secreted or if serum levels accurately reflect its activity within tumors.

In summary, ERO1α plays an important role in the occurrence and development of cardiovascular diseases, cancer, neurodegenerative diseases and other diseases. Exploring therapeutic strategies targeting ERO1α, such as the development of specific inhibitors and validation of ERO1α as a clinical biomarker, provides a new perspective for the diagnosis and treatment of these diseases.

## Glossary

ERO1α: Endoplasmic reticulum oxidoreductin 1α
PDI: protein disulfide isomerase
ROS: reactive oxygen
ERS: endoplasmic reticulum stress
ER: endoplasmic reticulum
FAD: flavin adenine dinucleotide
UPR: unfolded protein response
IRE1: inositolrequiring enzyme 1
PERK: protein kinase R (PKR)-like endoplasmic reticulum kinase
ATF: activating transcription factor
CHOP: C/EBP-homologous protein
SR: sarcoplasmic reticulum
SERCA2: sarco/endoplasmic reticulum Ca2+-ATPase2
GSSG: oxidized glutathione
GSH: reduced glutathione
RyR: ryanodine receptor
IP3R: inositol 1,4,5-trisphosphate receptor
EMT: epithelial-to-mesenchymal transition
ERp44: endoplasmic reticulum protein 44
STIM1: stromal interaction molecule 1
MAO: monoamine oxidase
I/R: ischemia/reperfusion
H/R: hypoxia/reperfusion
SIRT6: Sirtuin6
HIF1α: hypoxia-inducible factor 1α
HHcy: Hyperhomocysteinemia
T2DM: type 2 diabetes
STAT3: signal transducer and activator of transcription 3
VEGF: vascular endothelial growth factor
PD-L1: programmed cell death ligand 1
MPP+: 1-methyl-4-phenylpyridinium
SEPN1: Selenoprotein N
SEPN1-R: selenoprotein N-associated myopathy
TUDCA: tauroursodeoxycholic acid
HCV: Hepatitis C virus
LSD1: lysine-specific demethylase 1
